# Heat Transfer in Carbon-Nanotube Dispersions: A Simulation Study of the Role of Nanotube Morphology and Connectivity

**DOI:** 10.3390/molecules29204955

**Published:** 2024-10-19

**Authors:** Panagiota V. Polydoropoulou, Vasilis N. Burganos

**Affiliations:** Institute of Chemical Engineering Sciences, Foundation for Research and Technology-Hellas (FORTH/ICE-HT), Stadiou Str., Platani, P.O. Box 1414, GR26504 Patras, Greece; polydoropoulou@iceht.forth.gr

**Keywords:** nanofluids, carbon nanotubes, thermal conductivity, 3D numerical models

## Abstract

Simulation of the behavior of carbon nanotubes (CNTs) can become a very challenging task considering their complicated shape and large aspect ratio. This study aims to elucidate the role of CNT shape, length, and connectivity during heat transfer in CNT dispersions through a three-dimensional (3D) simulator. Three characteristic shapes for the CNTs are considered, namely, straight, moderately curved, and strongly curved. The results reveal that the commonly used assumption of viewing CNTs as straight cylinders leads to significant overestimation of the overall medium conductivity. The CNT length has an important effect on the nanofluid conductivity for all types of CNT shapes considered here. In addition, use of CNTs with higher conductivity than a certain value appears to have no further beneficial effect, thus relaxing the need for extremely pure or single-wall CNTs. On the contrary, the conductivity remains a strong function of the CNT concentration and may be even increased upon organization of CNTs into loose clusters. The overall approach and concept can be extended to CNT composites in a straightforward manner.

## 1. Introduction

The great advancement in material science and, more specifically, in nanotechnology has allowed engineers to develop innovative and more efficient multifunctional materials with the use of nanoparticles (NP) tailored to a multitude of engineering applications. During the last decades, nanofluids have emerged as a remarkably improved solution over conventional fluids for important heat transfer applications, for example, heat exchangers, heating and cooling devices, microelectronics, etc. [1]. Furthermore, the adoption of nanofluids can extend far beyond the state-of-the-art applications and holds promise for direct utilization in biomedical applications, tumor ablation, thermal management in batteries, thermoregulation textiles, solar collectors, etc.

In this context, a most promising candidate for use in advanced-technology applications is the class of carbon nanotubes (CNTs) thanks to their well-known, extraordinary properties [2], among which their excellent thermal conductivity. CNTs can play a substantial role in advanced nanofluids and nanocomposites by improving remarkably the heat transfer mechanisms of the base fluids or the polymer matrix, even at low volume fractions. As the CNTs are typically shaped as narrow, long, and waved tubes, it is anticipated that they will facilitate heat transfer by promoting phonon migration through longer paths and, thus, increase the thermal conductivity of the nanofluids. As the higher surface to volume ratio provides increased heat transfer, the shape of the nanoparticles appears to play a dominant role in the architecture of nanofluids and heat transfer. To maximize the effects of CNTs, careful selection of the major parameters has to be made, most notably the shape of the CNTs, the aspect ratio, the concentration, the diameter, the degree of agglomeration, and the interconnection paths. Experimental measurements have shown that the thermal conductivity of CNTs can vary over a wide range. In their ideal state without defects, their thermal conductivity can reach or even exceed in some cases, with the extraordinary value of 3000 W/mK. Therefore, in many theoretical studies, a variable value for the thermal conductivity of CNTs has been assumed in the absence of more accurate data.

Numerous experimental studies have been focused on the effect of CNTs on the thermal behavior of nanofluids. Thota et al. [3] found that the thermal conductivity increased by more than 20% upon addition of 0.1% *v*/*v* CNTs, where *v* stands for the volume. This was achieved by ensuring fine dispersion of CNTs at high stirring rates and long sonication times. Other studies [4,5] focused on eliminating CNT agglomeration during the preparation of the CNTs by controlling the sonication time. In Ref. [4], the results showed no agglomerates at long sonication times, yet the length of CNTs was significantly reduced. A high increase of 140% in the thermal conductivity of a nanofluid containing 2% *v*/*v* has been reported elsewhere [6]. To stabilize the sample, a surfactant was used, while the preparation of the mixture lasted more than 3 h. Omrani et al. [7] investigated the effect of size and shape of CNTs on the thermal conductivity by incorporating 0.05% *v*/*v* CNTs into distilled water. Six different types of CNTs were used. The authors found a maximum increase of 36% in the thermal conductivity of the nanofluids. They concluded that a fine dispersion of CNTs and an improved bonding of the nanoparticles with the base fluid by surface modification of the CNTs can lead to increased thermal conductivity. The rate of success in improving CNT-based nanofluids is a multiparameter concept based on the volume fraction, dispersion, temperature, aspect ratio, length, stability, etc. [3,4,5,6,7,8,9]. The maximum benefits of the CNTs have not been exploited up to now. The deep understanding of their behavior in suspensions will provide the knowledge to produce at high scales more stable, uniform nanofluids at low volume fractions, which will offer the maximum enhancement in thermal conductivity.

Computational modeling for the simulation of the effect of CNTs on the thermal behavior of nanofluids offers a valuable tool to investigate the role of the aforementioned parameters, given the huge number and cost of experiments that would be otherwise required. In fact, detailed characterization of nanofluids containing CNTs requires well-controlled conditions in order to elucidate the microscale mechanism of heat transfer and maximize the expected benefit. On the other hand, numerical simulators can greatly improve our understanding of the complicated mechanisms of heat conduction in nanofluids. Unfortunately, numerical studies on the effect of CNTs with regard to the thermal properties of nanofluids by conducting a multivariable analysis and considering the 3D waved shape of CNTs are dearth from the literature. Most numerical studies model CNTs as straight fibers [10] or spherical entanglements [11], or even as films and vertically aligned arrays [12]. Furthermore, the impact of CNT connectivity on heat transfer remains of strong interest to thermal behavior studies. In addition, the effect of the aspect ratio on the formation of connected CNT pathways and on the concomitant enhancement of the thermal conductivity of the nanofluids has not been sufficiently underlined.

This study attempts to elucidate and quantify the role of the most important parameters during heat transfer in nanofluids by developing an extensive 3D numerical simulation of CNTs and their organization into loose clusters in suspensions. A comprehensive 3D numerical study considering simultaneously all CNT major parameters as well as their clustering will address a major gap in the literature of heat transfer in nanofluids. The numerical results are compared to other theoretical and experimental results under the same conditions. It must be noted that CNTs are modeled in this study as fully 3D, waved tubes with cylindrical shape, built around an axis that is described by a spline function. These shapes are realistic representations of the ones that have been observed in electron microscopy images of CNTs in suspensions. Comparison with the simplified view of CNTs as straight tubes reveals and quantifies the strong effect of CNT shape on the medium conductivity. The possible overlapping effect of CNTs, especially at elevated volume fractions, has also been integrated into the simulator. It is evident that the simulator and results can be extended to CNT composites as well, in a straightforward manner.

## 2. Results and Discussion

The simulation results were compared to experimental data [4] of the effective thermal conductivities of nanofluids containing CNTs for various CNT lengths. It should be mentioned that the base fluid used in the experimental study has been distilled water (DW) with gum arabic (GA) as surfactant. The latter was completely dissolved in DW prior to the addition of CNTs and affected negligibly the conductivity of the fluid. Specifically, the effective thermal conductivity of the DW containing GA has been found to be only slightly less than the conductivity of pure DW (0.59 W/mK vs. 0.6 W/mK, respectively) [4]. The conductivity of the solution (DW and GA) has been used in the simulations that were carried out for validation purposes. The simulation results are also compared to the predictions of the analytical model that was based on Fricke’s expression [13] for spheroidal particles.

**Figure 1:**
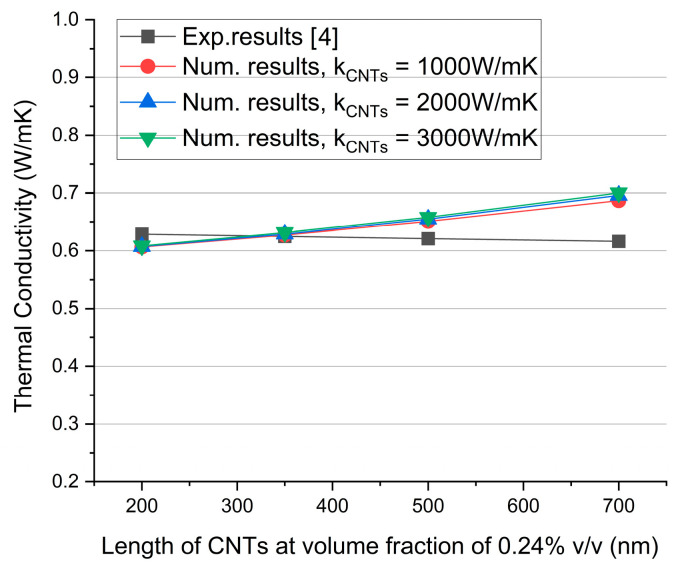
Graph of numerical and experimental results. The length of the CNTs is taken from SEM images provided in [4] and ranges from 200 nm to 700 nm. The diameter of the CNTs is 25 nm at a volume fraction of 0.24% *v*/*v*. The *k_CNT_* used in the numerical simulations ranges from 1000 W/mK to 3000 W/mK. The thermal conductivity of the base fluid was set to 0.59 W/mK, according to the measurements made in [4], where *K_CNTs_* is the thermal conductivity of CNTs.

Figure 1 shows the comparison of the simulations with experimental data [4] for different CNT lengths, as produced experimentally by varying the sonication time (between 10 and 40 min). Direct comparison with experiments is allowed here based on the previous discussion. In addition, three different values for the CNT conductivity are considered in this figure, covering a wide range of values that can lump the possible role of surfactants. The comparison with experiments is very good. It is noteworthy that moderately curved CNTs were used in the simulations to match similar shapes of CNTs as observed in SEM images [4]. The effect of CNT shape and quantified definitions of different levels of curviness are detailed in the next section. As the CNT length increases, the simulations predict a slight increase in thermal conductivity as a direct result of the elongated pathways for energy transfer along the CNT length. This is also in accord with the fact that phonon vibrations are the main mechanism for heat transfer in carbon nanotubes [14,15]. The ultrahigh thermal conductivity of CNTs originates from the vibration of the phonons along the tube. It should be noted that the mean free path (MFP) of phonons is based on an average value of all phonons’ MFPs. However, a large deviation between the mean value and the extreme values of the free path of phonons is expected. As a result, in reduced length of CNTs, a large number of phonons will not be able to contribute to heat transfer. In experiments, the conductivity appears to depend less on the CNT length as a result of the tradeoff between the effects of CNT length and agglomeration. More specifically, at reduced sonication times, CNTs are relatively longer, allowing for faster heat transfer, but also agglomerate stronger, resulting in reduced conductivity. The total outcome is a slight increase of the thermal conductivity by about 2% in the case of the shorter CNTs (200 nm) as compared to the longer CNTs (700 nm). The existence of the agglomerates has moderated the beneficial effect of the longer CNTs. But even at the maximum length of CNTs, the largest observed deviation of the numerical results from experiments was limited to only ~14%, which can be attributed mainly to the existence of CNT agglomerates.

**Figure 2:**
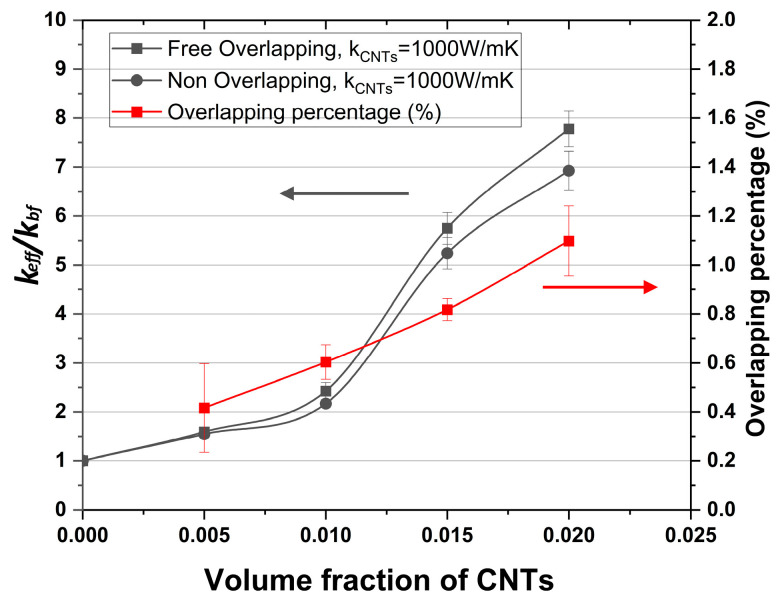
Dependence of nanofluid conductivity on the volume fraction of moderately curved CNTs. Effect of possible CNT overlapping. The left axis refers to the *k_eff_*/*k_bf_* ratio, whereas the right axis refers to the overlapping percentage of CNTs. k_CNT_ = 1000 W/mK, CNT length *L* =1 μm, and diameter *D* = 25 nm, where *k_eff_* is the effective thermal conductivity of the nanofluid and *k_bf_* is the thermal conductivity of the base fluid.

Figure 2 elucidates the effect of the volume fraction of CNTs on the thermal conductivity for overlapping and non-overlapping CNTs. The overlap indicates the common volume between the CNTs when no geometrical constraints are set during CNT placement. The overlapping percentage was calculated with respect to the total volume of CNTs in the RUC, as provided in Figure 2. Moderately curved CNTs are also used here, with a length of 1 μm. As it is expected, the impact of the overlapping effect becomes more pronounced at higher volume fractions. Yet, it should be noted that in both cases, a similar trend was predicted. At about a volume fraction of 1%, a sharp increase in the thermal conductivity is noticed. This can be attributed to the formation of connected pathways across the working sample in the direction of heat transfer and indicates the existence of a percolation threshold in this volume fraction region. Obviously, this value remains a function of the CNT length and, hence, cannot be defined uniquely.

**Figure 3:**
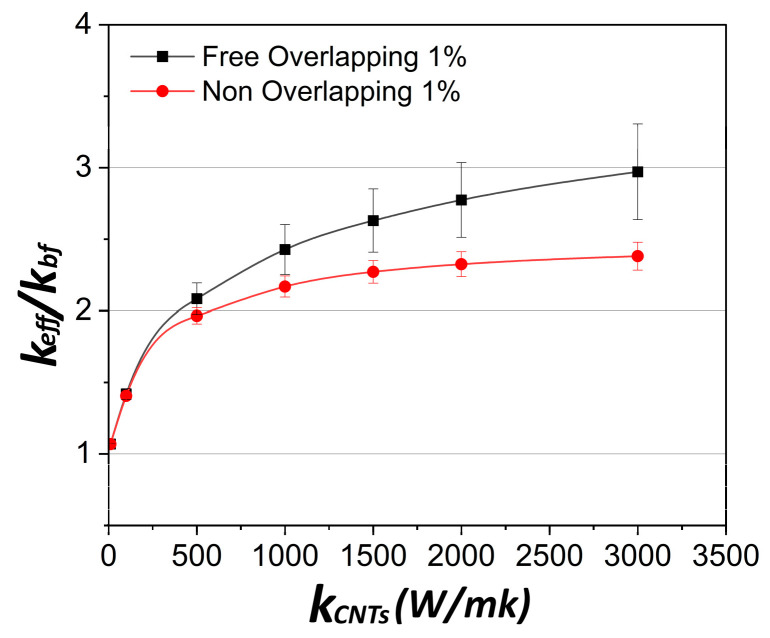
Dependence of the thermal conductivity of nanofluids on the CNT conductivity at 1% *v*/*v*. Effect of CNT overlapping. At volume fraction of 1% *v*/*v* of CNTs, the overlapping percentage of CNTs is 0.60%. CNT length *L* = 1 μm and diameter *D* = 25 nm.

The overlapping effect is also shown in Figure 3 as a function of the conductivity of individual CNTs, k_CNT_, keeping the volume fraction constant and equal to 0.01. For low values of k_CNT_, the overlapping effect is minimal. However, as k_CNT_ increases, this effect becomes progressively higher. More specifically, an increase of the thermal conductivity by ~25% is obtained for overlapping CNTs as compared to non-overlapping CNTs. This is a mere demonstration of the synergistic effect of connected CNTs on the nanofluid conductivity, which is absent in isolated CNTs. This observation suggests that controlled CNT agglomeration may improve the thermal behavior of the nanofluid under certain conditions, namely, when connected networks of CNTs are formed instead of CNT clustering into “clouds” without CNT contact. This point is further elaborated with additional simulations, as explained later in this section.

**Figure 4:**
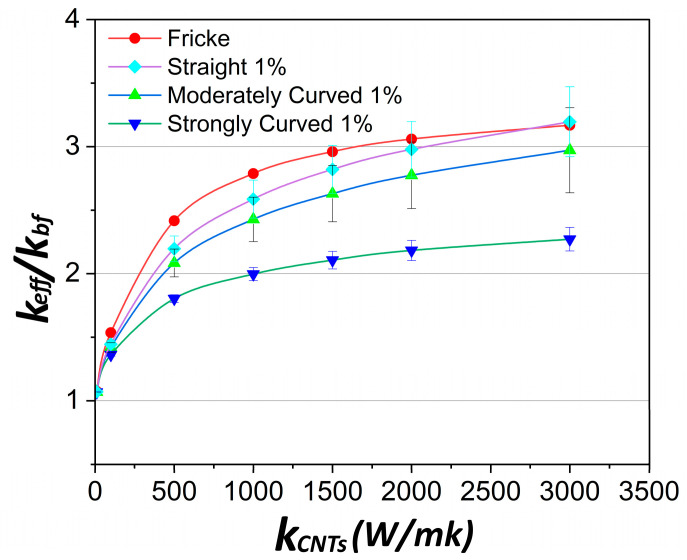
Effect of the shape of CNTs and correlation to the Fricke’s model at volume fraction of 1% *v*/*v*. Three different shapes of CNTs are used; more specifically, straight, moderately curved, and strongly curved CNTs. CNT length *L* = 1 μm and diameter *D* = 25 nm.

**Figure 5:**
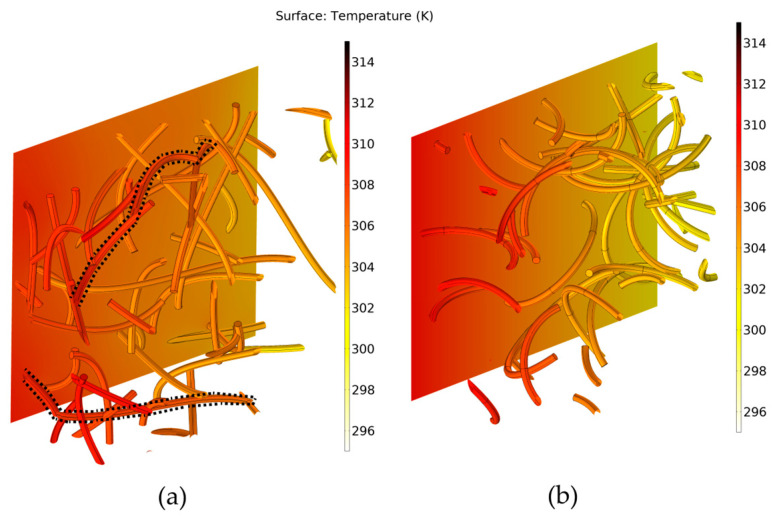
Temperature field (**a**) for moderately curved CNTs and (**b**) for strongly curved CNTs. Dotted lines highlight indicative nanotube paths across the structure along the heat transfer direction (volume fraction v = 1.5% *v*/*v*, *k_CNT_* = 3000 W/mK, CNT length *L* = 1 μm, and diameter *D* = 25 nm).

Figure 4 provides the numerical predictions results for the thermal conductivity under three different states of CNTs, namely straight, moderately curved, and strongly curved, keeping the CNT length constant and equal to 1 μm. As expected, straight CNTs offer the highest effective thermal conductivity. Fricke’s model predictions appear to correlate well with the simulation results for straight CNTs and tend to coincide with them at higher values of *k_CNT_*, namely, over 1500 W/mK. However, even at low bending stress, CNTs become waved [16]. As the degree of bending increases and CNTs become more curved, the effective thermal conductivity is remarkably decreased, as shown in Figure 4. This can also be noticed in Figure 5, which displays regions of moderately curved and strongly curved CNTs along with the numerically predicted temperature variation. Network paths are rarely formed in the case of the strongly curved CNTs, as they tend to form closed loops that fail to transfer heat down the temperature gradient. A second important observation is that above a specific value of k_CNT_, the value of the effective thermal conductivity tends to reach a plateau. The practical benefit of this result is that even moderate thermal conductivities of CNTs can be sufficient to achieve the same heat transfer enhancement as that by single-wall CNTs that are known to reach conductivity as high as 6000 W/mK.

**Figure 6:**
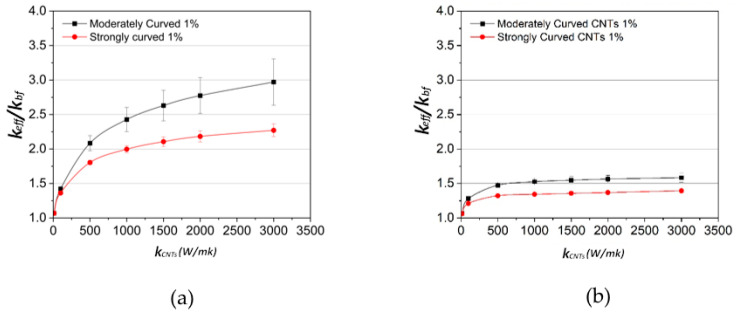
Dependence of nanofluid conductivity on k_CNT_ for moderately curved and strongly curved CNTs at volume fraction of 1% *v*/*v*: (**a**) CNT length *L* = 1 μm and (**b**) *L* = 500 nm (diameter *D* = 25 nm).

**Figure 7:**
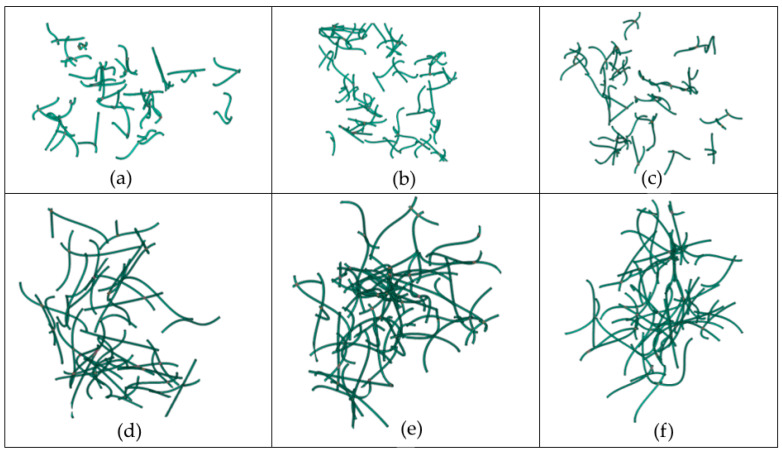
CNT networks at the same volume fraction (1% *v*/*v*) and different length sizes. Images (**a**–**c**) refer to the length of CNTs equal to 500 nm. Images (**d**–**f**) refer to the length of CNTs equal to 1 μm (diameter *D* = 25 nm).

The same observation can be made from Figure 6, which shows the dependence of the effective thermal conductivity of the nanofluid on the CNT conductivity and its variation with the CNT length and CNT curviness. The length of the CNTs appears to play a dominant role in heat transfer independently of the degree of CNT curviness. In fact, the results show that doubling the CNT length results in roughly double thermal conductivity of the nanofluid for high values of the CNT conductivity. This is noticed for both types of CNTs considered in this graph, namely, moderately curved and strongly curved CNTs. The effect of CNT curviness on the nanofluid conductivity becomes less pronounced as the CNT length decreases. From this point of view, some indicative images of networks connecting CNTs, with different lengths in each network, are provided in Figure 7. The networks were extracted from the Representative Unit Cells used in the current study and were developed by utilizing the commercial software COMSOL Multiphysics 6.0. Notice that the volume fraction in all images is the same and equal to 0.01. Images (a), (c), and (e) refer to length of CNTs equal to 500 nm; the rest of the images refer to length of CNTs equal to 1 μm. It is obvious that a denser network with better connectivity between CNTs is achieved in the case of the longer CNTs, which results in increased conductivity for longer CNTs. This can also be justified by the fact that phonons exceed the free mean length for longer CNTs, and hence, they efficiently contribute to the heat transfer [14,15,17,18].

A final remark is in order next, related not only to the effect of CNT morphology on the thermal conductivity of nanofluids but also to composite materials that contain CNTs. Given the increase in the thermal conductivity upon addition of CNTs, one can envisage those samples as loose clusters with very low volume fraction but with known thermal conductivity, as calculated using the present 3D simulator. The addition of such “clusters” to the main fluid, or matrix in the more general case, will eventually result in a medium that contains clusters with known conductivity. If the volume fraction of clusters in this medium is sufficiently small to allow application of the classical effective medium approximation, then one can predict the effective conductivity of the medium that contains several clusters or agglomerates with low volume fraction. The expression

*φ_CNT_* = *φ_A_*
*φ_CM_* (1)

can be used, where φ_CNT_ is the volume fraction of CNTs in the suspension, φ_A_ is the volume fraction of the clusters, and φ_CM_ is the volume fraction of clusters in the medium. Maxwell’s model can then be applied to the overall medium. As an alternative, fully 3D simulations were carried out at the scale of the overall medium, dispersing the aforementioned CNT clusters. This is particularly important for cases where φ_CM_ is larger than ~0.1, given the lack of accuracy of Maxwell’s expression for dense mixtures.

**Figure 8:**
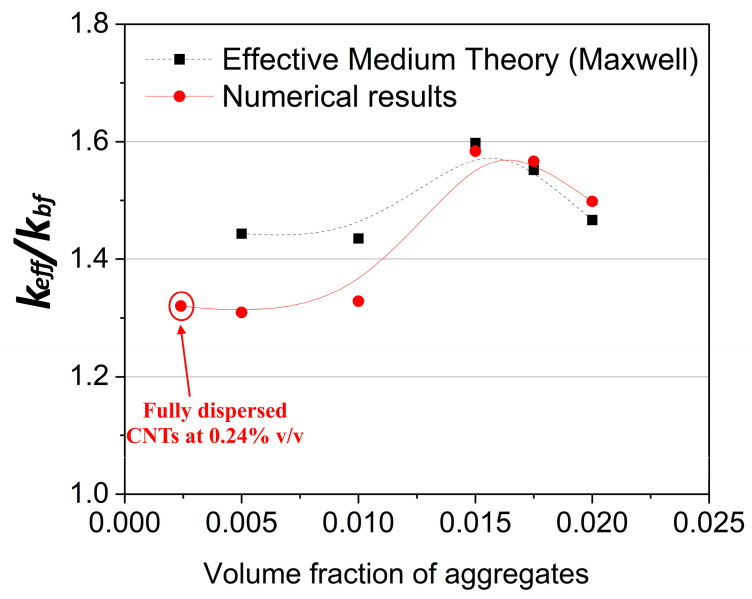
Thermal conductivity of a nanofluid containing CNT clusters compared to fully dispersed CNTs at a volume fraction of 0.24% *v*/*v*. *k_CNT_* = 2000 W/mK, CNT length *L* = 1 μm, and diameter *D* = 25 nm. The splines are used as guides to the eye.

The results are shown in Figure 8, where the thermal conductivity of a nanofluid containing CNT clusters is compared with the conductivity of a fully dispersed set of CNTs in a stable suspension. The numerical results are compared to Maxwell’s predictions quite satisfactorily for φ_CM_ below 15%, or equivalently, for φ_A_ higher than 1.5%. It is concluded that organization of the CNTs into loose clusters that are suspended in the medium may have a strongly beneficial effect on the thermal conductivity. As the volume fraction increases, the effective conductivity reaches a maximum of about 60% enhancement at a volume fraction of clusters equal to 1.5%. This is almost double enhancement of the nanofluid conductivity compared to that in the fully dispersed case (~30% enhancement compared to the base fluid) for the same overall volume fraction. Further investigation of this observation in a systematic fashion is already underway by the authors by extending the 3D simulator to account for the step-by-step agglomeration process and application of the numerical computations to the scale of the agglomerated medium as well.

## 3. Methods

The numerical analysis developed in this study includes a 3D detailed model of CNTs in a fluid medium aiming to predict the thermal conductivity by taking into account a number of key parameters. Validation is based on experimental results [4], whereas a comparison with the theoretical model of Fricke [13] is also conducted. It should be mentioned that the present numerical model investigates multiple parameters affecting the thermal conductivity of nanofluids. Yet, the simulation of agglomeration mechanisms and the role of surfactants is not included in this study as it is a special topic on its own and rests on the success and results of the present simulator.

The simulator starts with the placement of CNTs in the working domain, selecting representative shapes for the CNTs. As mentioned in the previous section, in most studies, CNTs are approximated as straight cylinders. Yet, the true shape of CNTs in suspensions after sonication differs significantly from that of a straight cylinder. CNTs are mostly met in a bent state as a result of possible defects in their lattice or external forces during production. Longer CNTs tend to become more curved as compared to shorter ones. Uniformly shaped CNTs are used in the present computations within a single realization, although it is clear that a mixture of different shapes can also be used in a straightforward manner. Three different shapes are selected for the present study, which are considered realistic following comparison with SEM images from the literature, as follows.

The shape of the CNTs is varied from straight cylindrical to strongly curved CNTs, including an intermediate shape that is moderately curved, as a result of the effect of pentagons and heptagons on their lattice leading to curved CNT structures [19]. More quantitatively, the moderately curved CNTs were simulated by a cubic spline passing through 4 points (*x*_0_, *y*_0_), (*x*_1_, *y*_1_), (*x*_2_, *y*_2_), and (*x*_3_, *y*_3_), as described by Equation (2) [20] as follows:

(2) $$g_{i}\left(x\right)=a_{i}{\left(x-x_{i}\right)}^{3}+b_{i}{\left(x-x_{i}\right)}^{2}+c_{i}\left(x-x_{i}\right)+d_{i}$$

i = 0, 1, 2, 3

where *a_i_*, *b_i_*, *c_i_*, and *d_i_* are parameters controlling the continuity and the shape of the curve. The 4 points were randomly selected following geometrical constraints. Specifically, the first point of the curve is randomly placed within the defined boundaries of the RUC. A new coordinate system x′,y′,z′ is established at this point with random orientation. The rest 3 points are placed on a fixed principal plane (for instance, z′ = 0) at random distances from the first point (within an arbitrary range). Specifically, the second point is within 15% of the total length, the second point between 20 and 30% of the total length, and the last one between 35 and 45% of the total length with regard to the first point. These are indicative values that reproduced realistic CNT geometries as observed from SEM images but can, obviously, be modified at will. The diameter of the CNTs is equal to 25 nm and the reference length is 1μm; comparison is provided also for CNT length equal to 0.5 μm. The condition that the total CNT length be equal to the prescribed length must, of course, be satisfied; otherwise, it is trimmed or discarded. Periodic conditions are applied at the CNT construction stage. Needless to say, other geometries and shapes can be used, provided some data from SEM pictures are available.

**Figure 9:**
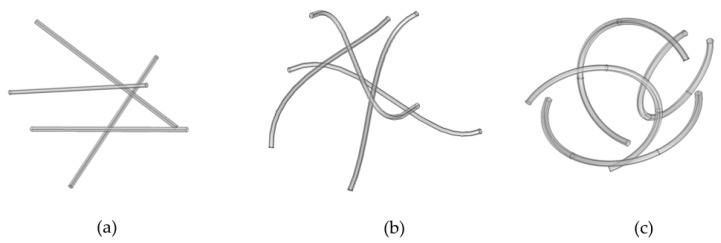
Indicative patterns of CNTs: (**a**) straight CNT, (**b**) moderately curved CNTs, and (**c**) strongly curved CNTs.

The strongly curved CNTs were simulated as arcs of flex angle in the range of ~[π–1.7π], which are compatible with several SEM images found in the literature, for example, [4]. The length is always constant and equal to the desired length. Figure 9 provides some indicative patterns of the generated CNTs.

**Figure 10:**
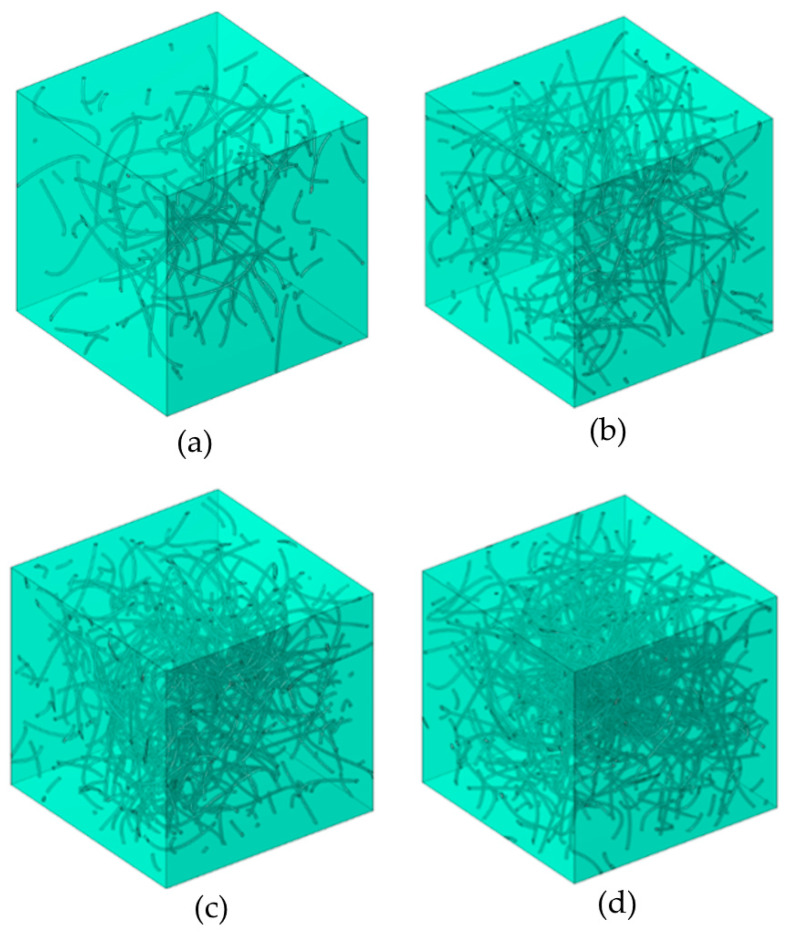
Representative Unit Cells of moderately curved CNTs. (**a**) 0.5% *v*/*v* CNTs, (**b**) 1.0% *v*/*v* CNTs, (**c**) 1.5% *v*/*v* CNTs, and (**d**) 2.0% *v*/*v* CNTs. (CNT length *L* = 1 μm and diameter *D* = 25 nm).

CNTs are gradually added to the working domain at random positions and with random orientation until the desired volume fraction is achieved. In the case of free overlapping, CNTs are added quite randomly, resulting in a possible mutual overlapping, which is typically quite limited due to the small diameter of the CNTs (25 nm). In the case of non-overlapping CNTs, a check is made whether the newly added CNT overlaps with any of the previously added CNTs, in which case the attempted position is discarded and a new position is selected. Upon conclusion, the algorithm leads to a structure that contains the prescribed volume fraction of CNTs with the desired option of CNT shape. Representative Unit Cells (RUCs), including randomly dispersed and randomly oriented CNTs, are shown in Figure 10. The RUC was sufficiently large to include CNTs of length 1 μm so as to account for potential interactions and synergies between neighboring particulates. Each RUC side was set to 2 μm. The volume fraction of the nanoparticles in the nanofluid was in the range of 0.005–0.02. The effects of volume fraction, shape, and length of CNTs and the overlapping between them can be quantified, as explained below.

**Figure 11:**
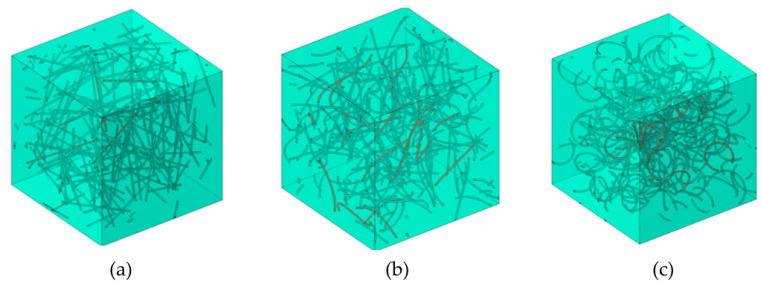
Representative Unit Cells of (**a**) straight CNTs, (**b**) moderately curved CNTs, and (**c**) strongly curved CNTs (volume fraction v = 1%, CNT length *L* = 1 μm, and diameter *D* = 25 nm).

Indicative samples of the whole geometry with respect to the curviness of the CNTs can be seen in Figure 11. The present simulations were implemented with the help of commercial software COMSOL Multiphysics 6.0.

**Figure 12:**
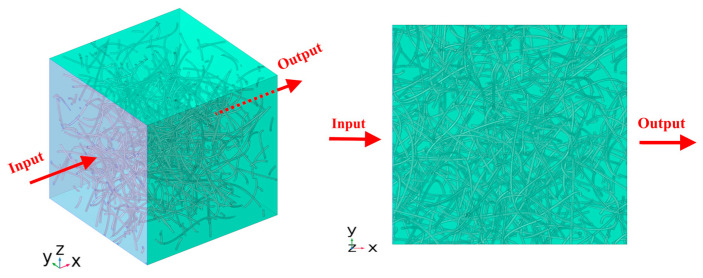
Temperature is fixed along the inlet face, and the outlet faces are fixed. The macroscopic heat transfer direction is indicated by the red arrow. The output of the heat flux is set on the opposite side along the x-direction. The rest sides are thermally insulated, with a heat flux q = 0.

At least 5 realizations for each case, including results in the three principal directions, were developed. For each realization, the thermal conductivity was calculated in all three directions x, y, and z using Dirichlet’s conditions on the inlet and outlet surfaces (fixed input and output temperature) and thermal insulation in the other two directions (Figure 12).

The mathematical equations that describe the problem are given below. As the heat transfer model describes a steady-state system without any external heat source, the solution for the 3D heat transfer model is provided by Equation (3) as follows:

(3) $$\left(k_{i}\nabla T_{i}\right)=0$$

where *k*_1,2_ and *T*_1,2_ are the conductivity and temperature in the domain of phase *i* = 1,2 (CNT or fluid), respectively.

In 3D Cartesian coordinates, for constant conductivity within an individual phase, Equation (3) gives the following equation:

(4) $$\frac{\partial^{2}T_{i}}{\partial x^{2}}+\frac{\partial^{2}T_{i}}{\partial y^{2}}+\frac{\partial^{2}T_{i}}{\partial z^{2}}=0$$

At the CNT-fluid interface, we assume smooth transition between phases, that is, continuity of temperature and continuity of the normal component of the heat flux as follows:

(5) $$T_{1}^{+}=T_{2}^{-}$$

and

(6) $$-k_{1}\nabla T_{1}^{+}\cdot \;\hat{n}=-k_{2}\nabla T_{2}^{-}\cdot \;\hat{n}$$

where $\hat{n}$ is the unit normal to the CNT–fluid interface.

The effective thermal conductivity was extracted from Fourier’s law at the domain scale, that is,

(7) $$q=-k_{eff}\nabla T$$

calculated at a fixed plane normal to the macroscopic heat flow direction. To increase accuracy and limit statistical errors, averages of this expression were taken over the entire working domain.

The mathematical equations, as described above, were solved by employing the Finite Element Method (FEM) utilizing COMSOL Multiphysics 6.0. Both the domain representing the fluid and the numerous smaller domains representing the CNTs were discretized into very small elements; detailed geometrical details are provided below. The mathematical equations are solved for each element, and then all solutions are merged by taking into account the boundary conditions to approximate the solution for the entire model. For the mesh of the domains, tetrahedral elements were chosen as they can be accurately adapted to complex geometries, as in the case of CNTs. Around sharp edges in the boundaries between the fluid and the CNTs, the mesh becomes extremely dense to avoid inaccuracies. The domain representing the fluid was meshed with elements of maximum size of about 40 nm and minimum element size of about 0.40 nm. For the domains of CNTs, 30 nm and 0.30 nm were used as the maximum and minimum element size, respectively. The above element sizes resulted in more than 100 million elements for the whole RUC cell for 1% *v*/*v* CNTs. For higher volume fractions, the number of elements was further increased. The number of elements was chosen after achieving mesh convergence.

Experimental measurements of the CNTs thermal conductivity have shown a wide range of values depending on the condition and possible defects of the CNTs [21,22]. Because of that, a wide range of values was used for the thermal conductivity of the CNTs in the simulations, namely, from 10 W/mK to 3000 W/mK.

For the sake of comparison with the effective medium approximation, a brief presentation of Fricke’s model [13] is given below, complemented with a new approach to allow better adaptation to the tubular shape of CNTs. Fricke’s model is described by Equation (8) as follows:

(8) $$k_{eff}=k_{bf}\frac{1+\frac{\beta \varphi }{1-\varphi }\frac{k_{np}}{k_{np}-k_{bf}}}{1+\frac{\beta \varphi }{1-\varphi }\frac{k_{bf}}{k_{np}-k_{bf}}}$$

where

*k_eff_* is the effective thermal conductivity of the nanofluid.
*k_bf_* is the thermal conductivity of the base fluid.
*k_np_* is the thermal conductivity of the nanoparticles.
*φ* is the volume fraction of CNTs within the fluid.

(9) $$\beta =\frac{1}{3}\left(\frac{k_{np}}{k_{bf}}-1\right)\left[\frac{2}{1+\frac{1}{2}\left(\frac{k_{np}}{k_{bf}}-1\right)M}\right]+\left[\frac{1}{1+\left(\frac{k_{np}}{k_{bf}}-1\right)(1-M)}\right]$$

with

(10) $$M=\frac{1}{{sin}^{2}x}-\frac{1}{2}\frac{{cos}^{2}x}{{sin}^{3}x}\ln\left(\frac{1+sinx}{1-sinx}\right)$$

and

(11) $$x=arccos\left(\frac{1}{R}\right)$$

*R* = *L*/*D*,

where *L* and *D* are the length and diameter of CNTs, respectively.

As the calculations of Fricke’s model are based on spheroids and not on cylinders, the geometry of the spheroids somehow differs from that of CNTs even for large aspect ratio values. To approximate better the case of CNTs, the average value of the diameter of the spheroid, <*D*>, was used in the calculations. For the calculations, an integration over the volume of the spheroid was made, described by Equation (12).

(12) D=ab∫0b1−y2b212dy

where *a* and *b* are the semiaxes of the spheroid (Equation (13)) as follows:

(13) $$\frac{x^{2}}{a^{2}}+\frac{y^{2}}{b^{2}}+\frac{z^{2}}{a^{2}}=1$$

The value of the average diameter was calculated analytically to equal (*π*/*4*)*D. The predictions of the effective medium approximation, adapted as above, are compared to the numerical simulation results in the next section.

## 4. Conclusions

The effect of CNTs on heat transfer in nanofluids was investigated by means of a 3D simulator that paid particular attention to the morphology of the medium with special emphasis on the CNT shape. A parametric numerical analysis taking into account the length, the shape, the volume fraction, the thermal conductivity, and the overlapping effect was performed. Experimental observations and measurements are reproduced quite satisfactorily by the present simulator.

A rapid increase in the effective thermal conductivity is predicted at the volume fraction of about 0.01 for CNT length 1μm. This indicates the formation of percolating clusters of CNTs, spanning the working sample. The exact value of the percolation threshold mostly depends, however, on the length of the CNTs. Longer CNTs promote more efficient heat transfer in nanofluids.

The commonly used assumption in literature models that CNTs are straight cylindrical tubes was shown here to overestimate significantly the actual thermal conductivity of the nanofluid. The present simulations quantify the reduction in the nanofluid conductivity for progressively curved CNTs and are aligned with the experimental observation that the enhancement of the nanofluid conductivity is less drastic than anticipated. These results can be directly transferred to composite materials that contain CNTs as fillers, including not only polymers with improved mechanical or thermal properties but also mixed matrix membranes for water treatment, and elucidate the role of CNT shape on the properties of the composite.

It is also noteworthy that an increase in the CNT conductivity beyond a certain value, around 2000 W/mK, has a limited effect on the nanofluid conductivity, in accord with relevant predictions of the effective medium theory, especially for the lengthier type of CNTs examined here (1 μm). This is an important result as it relaxes the need for ultrapure or single-wall CNTs that offer extreme thermal conductivities, that is, beyond 2000–3000 W/mK.

Organization of CNTs into loose clusters (volume fraction in the range of 1–2%) and dispersion of the clusters in the base fluid have resulted in a clearly beneficial effect on the thermal conductivity of the medium. In fact, at a certain level of the cluster compactness (expressed by the volume fraction inside the cluster), a maximum value of the thermal conductivity increase was obtained that was almost double that in the fully dispersed case. This suggests that further improvement in the thermal conductivity of CNT-based nanofluids can be obtained, depending on the internal organization of the CNTs into clusters or agglomerates.

The above considerations can serve as valuable guidelines for the efficient production of tailored nanofluids or even composite materials. For appropriate architectures, CNTs can provide faster heating or cooling and advance the conventional approaches that are currently used.

## Data Availability

Data availability upon reasonable request.
